# Massive intraocular hemorrhage, presumably from the central retinal artery after cataract surgery, and difficult hemostasis during vitrectomy: a case report

**DOI:** 10.1186/s12886-022-02555-z

**Published:** 2022-08-08

**Authors:** Suguru Nakagawa, Kiyoshi Isii

**Affiliations:** Department of Ophthalmology, Saitama Red Cross Hospital, 1-5, Shintoshin, Chuo-ku, Saitama, Japan

**Keywords:** Cataract surgery, Massive intraocular hemorrhage, Central retinal artery, Intraocular lens dislocation, General anesthesia, Difficult hemostasis

## Abstract

**Background:**

Severe intraocular hemorrhage is a rare complication of cataract surgery due to the recent generalization of minimal-incision cataract surgery. We report a case of a massive intraocular hemorrhage that probably originated from the central retinal artery after cataract surgery, in which hemostasis was difficult to achieve during vitrectomy.

**Case presentation:**

An 86-year-old woman was referred to our department for intraocular lens (IOL) dislocation after undergoing cataract surgery. Massive intraocular hemorrhage was observed during the initial visit to our department. She underwent pars plana vitrectomy (PPV) and IOL repositioning under local anesthesia. However, the hemorrhage could not be removed completely because of continued massive intraoperative bleeding from the posterior fundus, and it was extremely difficult to achieve hemostasis during the initial surgery. At 7 days after the initial surgery, PPVs were performed under general anesthesia. Bleeding significantly decreased in the second surgery compared to the first. The bleeding probably originated from the central retinal artery on the optic disc; hemostasis was obtained by coagulation of the bleeding site with intraocular diathermy. After the second surgery, there was no exacerbation of bleeding and the patient’s condition was stable. However, the patient’s visual acuity showed no light perception after the second surgery.

**Conclusions:**

Massive intraocular hemorrhage may occur from the central retinal artery after undergoing cataract surgery. In such cases, surgery with general anesthesia with a lower maintained blood pressure (instead of surgery under local anesthesia) should be recommended, considering the possibility of difficult hemostasis in the event of bleeding from the retinal artery.

**Supplementary Information:**

The online version contains supplementary material available at 10.1186/s12886-022-02555-z.

## Background

Hemorrhagic complications of intraocular surgery, including cataract surgery, comprise anterior chamber, vitreous [1, 2], and expulsive hemorrhage [3]. Recently, because of the growing popularity of phacoemulsification with a small incision, serious hemorrhagic complications have become rare. This case report presents a surgical case in which massive intraocular hemorrhage was observed after extracapsular cataract extraction (ECCE), intraocular lens (IOL) implantation, and repositioning of the dislocated IOL for a mature cataract at a local clinic. The massive intraocular hemorrhage and the continued bleeding from the posterior pole led to poor visibility, and the bleeding site could not be identified accordingly; in particular, it was extremely difficult to achieve hemostasis during the initial vitrectomy under local anesthesia. During the second vitrectomy under general anesthesia, we observed bleeding that had probably originated from the central retinal artery on the optic disc, and hemostasis was finally achieved.

## Case presentation

The ethics committee at our institution waived the requirement of approval for this retrospective case report. All procedures conducted in this study adhered to the tenets of the Declaration of Helsinki. Written informed consent was obtained from the patient.

**Fig. 1:**
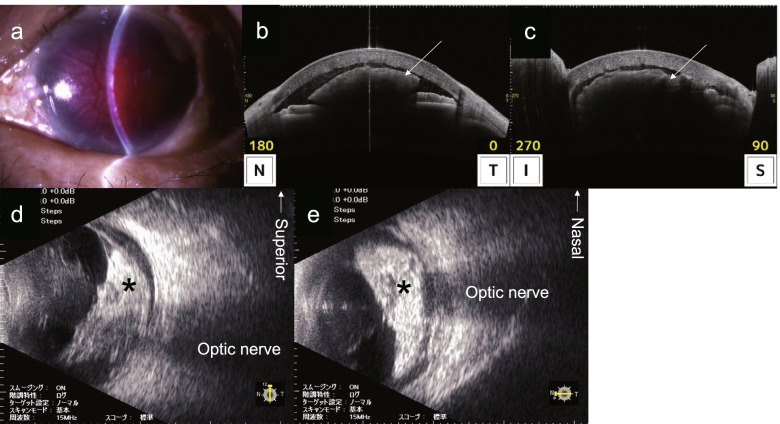
Massive intraocular hemorrhage at the initial visit to our department. Slit-lamp examination (**a**) and anterior segment optical coherence tomography (**b**, **c**) revealing corneal edema and massive hyphema (arrow) in the anterior chamber. A hyperechoic image indicative of vitreous hemorrhage is observed on ocular ultrasound examination (**d**, **e**). No evident findings of retinal detachment and choroidal detachment are observed

An 86-year-old woman underwent ECCE for a mature cataract in her left eye at a local clinic. As posterior capsular rupture was observed during the ECCE, a three-piece acrylic IOL with an optic diameter of 7.0 mm (X-70, Eternity®, Santen Co., Ltd., Osaka, Japan) was implanted in the sulcus. The next day, IOL dislocation was observed; therefore, repositioning of the IOL was attempted, which resulted in a complete IOL dislocation into the posterior chamber. Subsequently, the patient was referred to our department on the same day. Whether there was intraocular hemorrhage at the time of referral remains unknown; however, there was no mention of intraocular hemorrhage in the referral letter from the previous doctor to our department. Hence, there was a high possibility of no bleeding. At the initial visit to our department, her best-corrected visual acuity was 20/25 in the right eye and perception of light (±) in the left eye, while the intraocular pressure (IOP) was 13 and 17 mmHg in her right and left eye, respectively. Slit-lamp examination and anterior segment optical coherence tomography revealed massive hyphema in the left eye (Fig. 1 a–c). The IOL and fundus could not be observed because of poor visibility caused by corneal edema and massive intraocular hemorrhage. Ocular ultrasound revealed a hyperechoic image which appeared to be a vitreous hemorrhage (Fig. 1 d, e). She had undergone cataract surgery in her right eye at a local clinic 6 months ago. Only drusen and microaneurysms were observed on the fundus of the right eye. No other lesions (such as retinal hemorrhage or exudates) indicative of diabetic retinopathy (DR) or other ischemic ocular diseases were found in the right eye (Additional file 1). Furthermore, no lesions indicative of DR or other ischemic ocular diseases were observed in the fundus of the left eye before cataract surgery during examination by the previous physician. She had type 2 diabetes and hypertension that were treated by a local physician. However, a blood test at our department revealed a hemoglobin A1c level of 9.9%, indicating poor glycemic control. She had not been taking anticoagulants and antiplatelet drugs, and the preoperative blood tests revealed no abnormal values for the coagulation function. The axial lengths were 22.89 and 22.86 mm in her right and left eye, respectively, and an IOL was fixed in the capsular bag in the right eye. She was immediately hospitalized for glycemic control at the Department of Diabetic Medicine; at 5 days after her initial visit to our department, she underwent a pars plana vitrectomy (PPV) and IOL repositioning under local anesthesia.

### Initial intraoperative findings

**Fig. 2:**
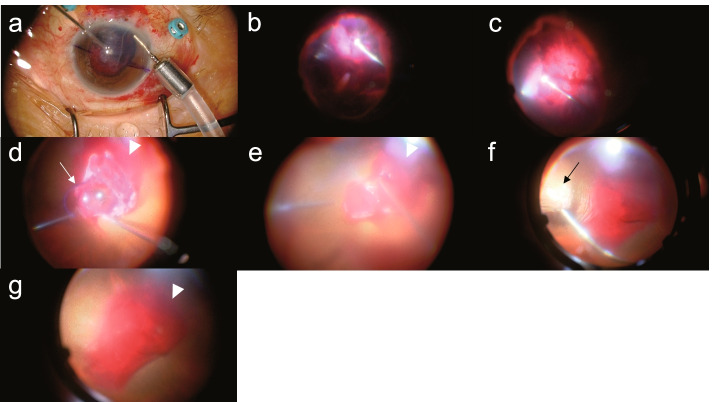
Intraoperative findings in the initial surgery. Massive hyphema in the anterior chamber removed using a 25-gauge vitreous cutter (**a**). Massive vitreous hemorrhage is observed (**b**, **c**). As the removal of hemorrhage progresses (**b**, **c**), the intraocular lens is found in the hemorrhage of the posterior fundus (**d**; arrow); simultaneously, massive bleeding is observed from the vicinity of the optic disc (**d**, **e**, **g**; the arrowhead shows the bleeding site). Hemostasis is attempted by elevating the intraocular pressure and aspirating the blood using a 25-gauge cutter; however, it was difficult to achieve. Moreover, it was difficult to identify the bleeding site and coagulate with intraocular diathermy. The retina was pale at the temporal side (**f**; arrow), suggesting retinal ischemia because of impaired retinal circulation attributed to damage of the retinal artery. Retinal photocoagulation performed at the temporal side of the retina (**f**, arrow). The optic disc and macula cannot be observed until the end of the surgery (**g**) because of hemorrhage

After retrobulbar anesthesia, a 25-gauge vitrectomy system (Constellation® Vision System, Alcon Laboratories, Inc., Fort Worth, TX) and wide-angle viewing system (Resight® Fundus Viewing System, Carl Zeiss Meditec AG, Jena, Germany) were used for the surgery. First, the massive hyphema in the anterior chamber was removed using a 25-gauge vitreous cutter (Fig. 2a). Furthermore, while the massive vitreous hemorrhage was removed (Fig. 2b, c), the IOL could be seen in the hemorrhage of the posterior fundus and appeared to be present above the optic disc (Fig. 2d). Although the hemorrhage had decreased, it could not be removed completely due to poor visibility secondary to the massive bleeding in the posterior fundus (Fig. 2d, e, g). Despite the temporary improvement in visibility when the blood was aspirated using a 25-gauge cutter, the vitreous cavity immediately filled with blood again, resulting in poor visibility. Hemostasis could not be achieved by the transient elevation in intraocular pressure (IOP), which was performed intraoperatively. Coagulation with intraocular diathermy was also considered; however, it was difficult to identify the bleeding site because of poor visibility. The macula and optic disc could not be observed throughout the surgery owing to hemorrhage (Fig. 2g). The temporal side of the retina was pale, suggesting retinal ischemia due to impaired retinal circulation secondary to retinal artery damage. Photocoagulation was performed on the temporal side of the retina (Fig. 2f), followed by repositioning of the dislocated IOL in the sulcus. The intraoperative blood pressure (BP) was 155/82 mmHg at the beginning of surgery. The intraoperative systolic BP (SBP) was high at 174.9 ± 15.3 mmHg (mean ± standard deviation), with the peak SBP at 207 mmHg; the intraoperative diastolic BP (DBP) was high at 86.3 ± 12.2 mmHg, with the peak DBP at 145 mmHg. Despite two intravenous injections of nicardipine during the surgery, the BP remained high. The BP at the end of the surgery was 207/83 mmHg (SBP/DBP).

### Postoperative course after the initial surgery

**Fig. 3:**
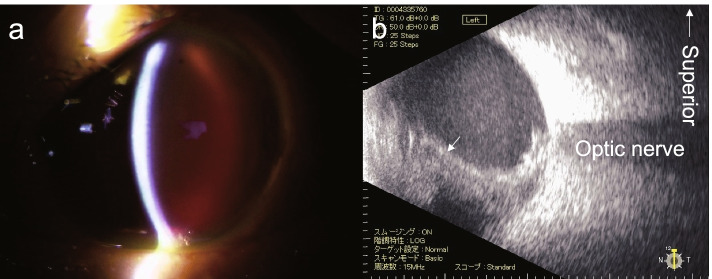
Intraocular hemorrhage after the initial surgery. Bleeding in the anterior chamber (**a**). Hyperechoic image of vitreous hemorrhage during ocular ultrasound examination (**b**, arrow)

After the initial surgery, the intraocular hemorrhage worsened again (Fig. 3); at 7 days postoperatively, encircling, PPV, and silicone oil tamponade were scheduled under general anesthesia.

### Second intraoperative findings

**Fig. 4:**
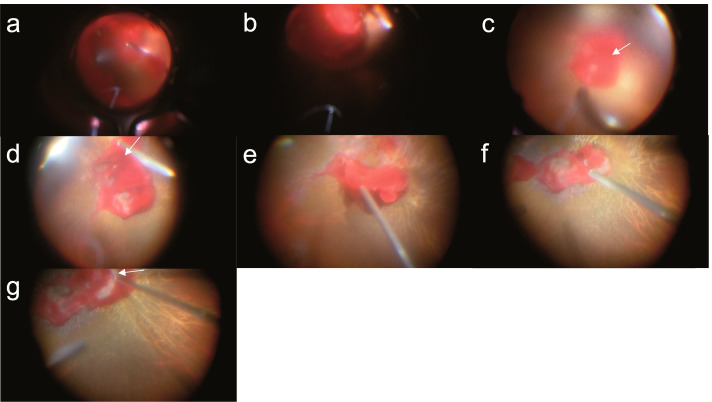
Intraoperative findings in the second surgery. As the removal of the massive hemorrhage progresses (**a**, **b**), bleeding from the vicinity of the optic disc is observed (**c**, **d**, **g**; arrow). The amount of bleeding is significantly lower than that in the first surgery, and the intraoperative visibility is significantly better. Hemostasis is achieved by aspirating the blood under transiently elevated intraocular pressure (**e**, **f**) and coagulating with intraocular diathermy (**g**, arrow)

After general anesthesia, 360-degree scleral buckling was performed using a silicone circling band (#240, Mira Inc., Uxbridge, MA). Then, the massive hyphema in the anterior chamber was removed using a 25-gauge cutter. Bleeding from the posterior fundus was significantly lower in the second surgery than in the first under local anesthesia. As the removal of the massive hemorrhage progressed (Fig. 4a, b), bleeding was observed from the nasal side of the optic disc, which probably originated from the central retinal artery on the optic disc (Fig. 4c, d and presented from 1 min 45 s after the start of the video in Additional file 2). Hemostasis was then achieved by a transient elevation in IOP, aspiration of blood using a 25-gauge cutter (Fig. 4e, f), and coagulation of the bleeding site with intraocular diathermy (Fig. 4g, and 2 min 40 s after the start of Additional file 2), followed by silicone oil tamponade. Paleness of the optic disc was observed during surgery. The intraoperative SBP and DBP were stable at 124.7 ± 11.2 mmHg (peak SBP: 172 mmHg) and 60.5 ± 3.6 mmHg (peak DBP: 71 mmHg), respectively.

### Postoperative course after the second surgery

**Fig. 5:**
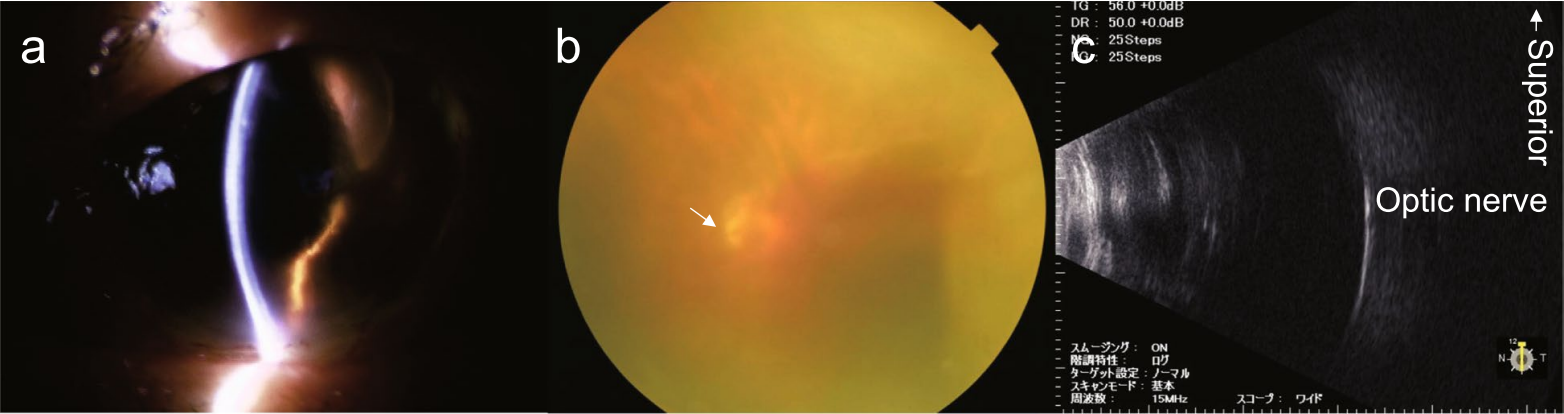
Postoperative findings at 7 days after the second surgery. No bleeding is observed in the anterior chamber during a slit-lamp examination (**a**). The optic disc and coagulation site are observed in the posterior fundus (**b**). Ocular ultrasound examination does not show any hyperechoic images suggestive of vitreous hemorrhage (**c**)

After the second surgery, there was no exacerbation of bleeding, and the patient’s condition was stable (Fig. 5). The patient’s visual acuity showed no light perception 7 days after the second surgery.

## Discussion and conclusions

Vitreous hemorrhage is a relatively common complication of conventional cataract surgeries, such as ECCE and intracapsular cataract extraction [1]; however, bleeding from the central artery is rare in these conventional surgeries. Furthermore, bleeding is a rare complication of newer cataract surgeries, such as small-incision phacoemulsification [1, 2, 4]. The causes of vitreous hemorrhage after cataract surgeries have been reported to be wound bleeding [5, 6], iris bleeding after an iris resection, and vascular rupture of the ciliary body and peripheral retina caused by zonular traction [1].

Herein, we report a case of a massive intraocular hemorrhage after cataract surgery, in which hemostasis was notoriously difficult to achieve. The bleeding site was identified on the nasal side of the optic disc in the second vitreous surgery under general anesthesia, and hemostasis was achieved thereafter. The most common cause of bleeding from the vicinity of the optic disc is bleeding from neovascularization of the disc (NVD). However, in the present case, there were several findings that indicated that the bleeding originated from the central retinal artery and not from NVD. First, the bleeding site did not appear to arise from the optic disc and did not exhibit the characteristics of NVD (which grows with the posterior vitreous face as the scaffold). Hence, direct diathermy to the bleeding sites over the optic disc could not be avoided for obtaining hemostasis. However, there was a limitation in that the intraoperative video was not centered in the area of active bleeding. Second, during the first vitrectomy, bleeding continued even though the IOP increased transiently and the blood was aspirated; thus, it was impossible to identify the hemostatic point. In case of bleeding from NVD, we could have reduced the amount of bleeding by temporarily increasing the IOP, identified the bleeding point, and obtained hemostasis by diathermy coagulation. The present case involved massive intraocular bleeding (which we had never encountered before), and it was extremely difficult to identify the bleeding point and achieve hemostasis. Third, no fundus lesions that could cause NVD, such as those indicative of DR or ischemic ocular diseases, were observed in the affected or fellow eyes before the cataract surgeries by the previous physician. Fourth, no significant carotid artery occlusion, which could cause the ocular ischemic syndrome and lead to NVD, was identified in the bilateral carotid arteries after performing a carotid Doppler test. Lastly, no iris rubeosis or increased ocular pressure suggestive of the ocular ischemic syndrome were observed. Based on these findings, the possibility of bleeding from NVD was low in this case, and we concluded that the bleeding originated from the central retinal artery on the optic disc. Paleness of the retina and the optic disc, which may be observed in the ocular ischemic syndrome, were observed during the vitrectomies in the present case. However, this paleness may have been caused by retinal ischemia, which was attributed to an impaired retinal circulation secondary to bleeding from the central retinal artery. Although the possibility of bleeding from NVD cannot be ruled out completely, we believe that bleeding from the central retinal artery was more likely in the present case.

However, in this case, the perception of light was lost after the second vitrectomy. This could be attributed to the fact that ocular ischemia was associated with bleeding from the central retinal artery; intraoperative findings may reveal paleness of the optic disc and retina, which are attributed to secondary ocular ischemia following bleeding from the central retinal artery. The other possible cause was the direct damage to the optic nerve due to diathermy coagulation. Due to bleeding from the central retinal artery on the optic disc, direct diathermy at the bleeding sites over the optic disc could not be avoided to achieve hemostasis; this procedure can cause a loss of light perception. In contrast, in cases of bleeding from NVD, diathermy away from the optic disc can prevent a loss of light perception.

In this case, the central retinal artery (and not the peripheral retinal blood vessels as previously reported [1]) was presumed to be damaged. To the best of our knowledge, there are no reports of bleeding from the central retinal artery as the cause of intraocular hemorrhage after cataract surgery. It remains unclear why the damage to the central retinal artery occurred, as this cataract surgery was performed by another physician. As the IOL had dislocated near the bleeding site, it was possible that the use of a larger IOL (7.0 mm) with rigid haptics made of polyvinylidene difluoride may have been associated with the central retinal artery damage, indicating the possibility of mechanical damage by the IOL to the optic disc vasculature.

Suprachoroidal and expulsive hemorrhage are other severe hemorrhagic complications of cataract and intraocular surgeries [3, 7]. The contents of the eye are expelled, especially in case of expulsive hemorrhage, thereby leading to blindness. Moreover, it is important to consider suprachoroidal and expulsive hemorrhage as possible causes of severe hemorrhagic complications; however, in the present case, no choroidal ridge characteristics of suprachoroidal and expulsive hemorrhage were observed before or during surgery.

Perioperative hypertension may increase the risk of blood loss, myocardial ischemia, and cerebrovascular events [8]. Although the BP during the initial surgery under local anesthesia was high at 175/86 mmHg (SBP/DBP), the BP during the second surgery under general anesthesia was low at 125/61 mmHg. Consequently, the second surgery had significantly reduced bleeding [8] and an improved intraoperative visibility; thus, hemostasis was achieved by intraocular diathermy coagulation of the bleeding site. It is important to maintain a low perioperative BP to reduce bleeding [8]. Therefore, surgery under general anesthesia by an anesthesiologist, and not under local anesthesia by an ophthalmologist, should be recommended in cases of severe arterial bleeding, as in the present case.

In conclusion, we encountered a rare massive intraocular hemorrhage, which probably originated from the central retinal artery after cataract surgery. For massive intraocular hemorrhage after an intraocular surgery, considering the potential for arterial bleeding, local anesthesia by an ophthalmologist may be insufficient to control the BP and stop the hemorrhage; in such cases, surgery with general anesthesia, wherein the BP is controlled by an anesthesiologist, should be considered.

## Supplementary Information

**Additional file 1.** Fundus findings of the fellow eye. Fundus photograph (a, b), fluorescein angiography (c, d) and optical coherence tomography (e) of the fellow eye revealing only drusen and microaneurysms. No other lesions (such as retinal hemorrhage or exudates) indicative of diabetic retinopathy (DR) or other ischemic ocular diseases were found.

**Additional file 2.** The surgical video of the 2nd vitrectomy. Arterial bleeding was aspirated from the nasal side of the optic disc (this information is presented from 1 min 21 s after the start of the video), but bleeding originated from the nasal side of the optic disc again (this information is presented from 1 min 45 s after the start of the video). Intraocular diathermy coagulation of the nasal side of the optic disc, which appeared to be the site of bleeding, was performed (this information is presented from 2 min 40 s after the start of the video).

## Data Availability

All data supporting the findings of the present study are contained within the manuscript (and its additional files).

## Abbreviations

ECCE: Extracapsular cataract extraction
IOL: Intraocular lens
IOP: Intraocular pressure
PPV: Pars plana vitrectomy
BP: Blood pressure
SBP: Systolic blood pressure
DBP: Diastolic blood pressure
DR: Diabetic retinopathy
NVD: Neovascularization of the disc
