# Correction: Massive intraocular hemorrhage, presumably from the central retinal artery after cataract surgery, and difficult hemostasis during vitrectomy: a case report

**DOI:** 10.1186/s12886-022-02588-4

**Published:** 2022-09-07

**Authors:** Suguru Nakagawa, Kiyoshi Ishii

**Affiliations:** Department of Ophthalmology, Saitama Red Cross Hospital, 1-5, Shintoshin, Chuo-ku, Saitama, Japan

**Correction: BMC Ophthalmol 22, 336 (2022)**

**https://doi.org/10.1186/s12886-022-02555-z**

Following the publication of the original article [1], we were notified of a spelling mistake in the second author’s name.

Originally published name: Kiyoshi Isii

Corrected name: Kiyoshi Ishii

The original article has been corrected.
