# Supplementary figures and images for: Massive intraocular hemorrhage, presumably from the central retinal artery after cataract surgery, and difficult hemostasis during vitrectomy: a case report
Source: BMC Ophthalmol. 2022 Aug 8;22:336. doi: 10.1186/s12886-022-02555-z (PMC9358848; doi:10.1186/s12886-022-02555-z)

# Additional file 1

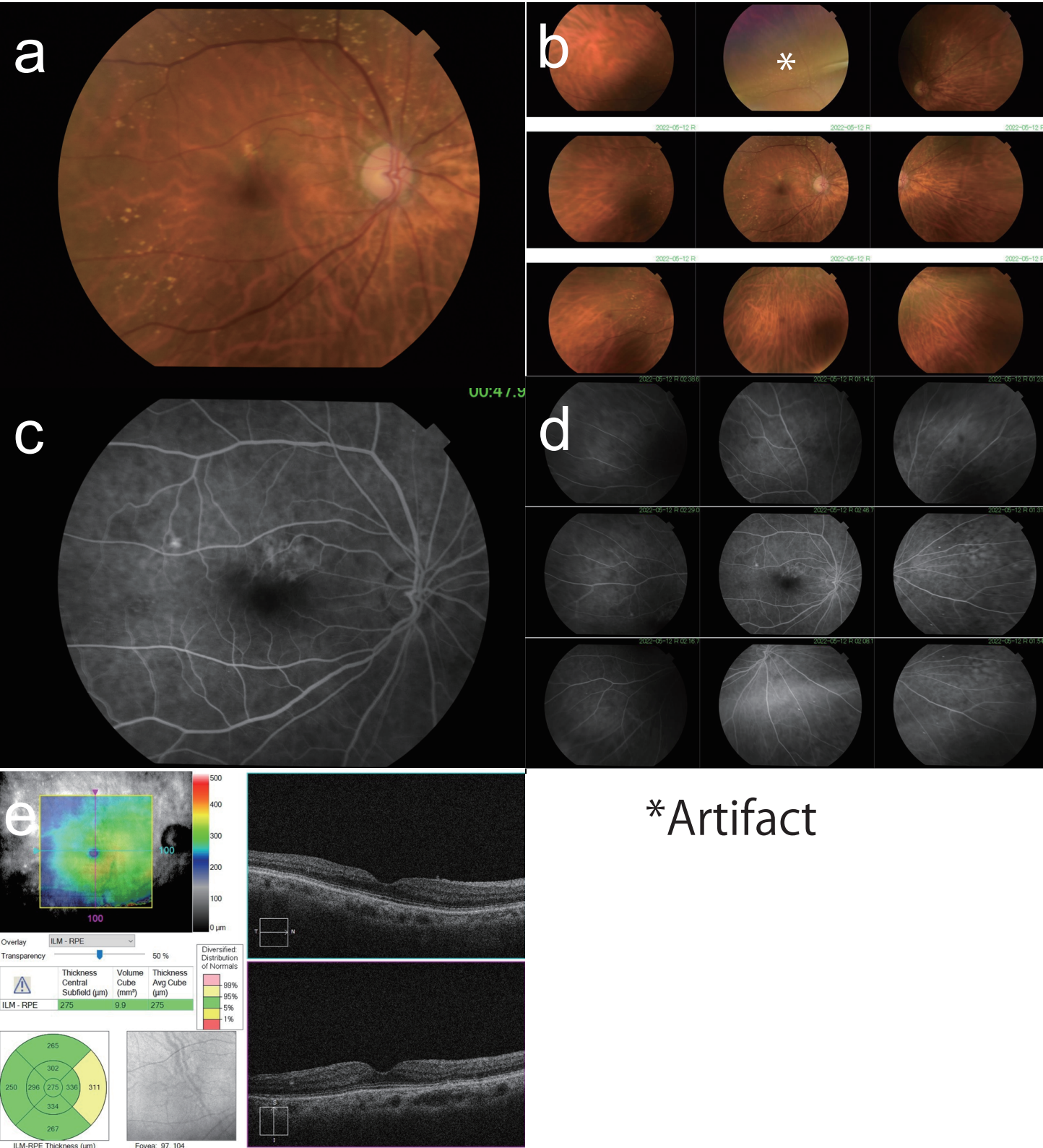

Supplement: Supplementary file 1 — Additional file 1. Fundus findings of the fellow eye. Fundus photograph (a, b), fluorescein angiography (c, d) and optical coherence tomography (e) of the fellow eye revealing only drusen and microaneurysms. No other lesions (such as retinal hemorrhage or exudates) indicative of diabetic retinopathy (DR) or other ischemic ocular diseases were found. [file 12886_2022_2555_MOESM1_ESM.pdf]
